# Arthritis in children with LRBA deficiency – case report and literature review

**DOI:** 10.1186/s12969-019-0388-4

**Published:** 2019-12-17

**Authors:** Rotem Semo Oz, Melissa S. Tesher

**Affiliations:** Section of Pediatric Rheumatology, University of Chicago Medical Center, 5841 South Maryland Avenue, Room C101, MC, Chicago, IL 5044 USA

**Keywords:** LRBA deficiency, Arthritis, Juvenile idiopathic arthritis, Review

## Abstract

**Background:**

Lipopolysaccharide (LPS)-responsive and beige like anchor (LRBA) deficiency is categorized as a subtype of common variable immune deficiency (CVID). A growing number of case reports and cohorts reveal a broad spectrum of clinical manifestations and variable phenotype expression, including immune dysregulation, enteropathy and recurrent infections. The association between rheumatic disease and CVID generally has been well established, arthritis has been less frequently reported and minimal data regarding its clinical features and characteristic in LRBA deficiency has been published. This case report and literature review evaluates the characteristics and features of arthritis in LRBA deficiency patients.

**Case presentation and review results:**

Herein, we describe a unique case of LRBA deficiency first presented with poly articular arthritis. Alongside the report, a literature review focusing on LRBA deficiency, rheumatic disease and arthritis has been conducted. We reviewed 43 publications. Among these, 7 patients were identified with arthritis. Age of first presentation was six weeks to 3 years. Male to female ratio was 4/3. Two patients were diagnosed with polyarticular Juvenile idiopathic arthritis (JIA) and three with oligoarticular JIA. Each patient was found to have different genomic mutation. The treatment was diverse and included corticosteroids, cyclosporine, methotrexate, adalidumab and abatacept.

**Conclusion:**

Joint involvement is variable in LRBA deficiency, hence it should always be kept in mind as a differential diagnosis for a patient with combination of juvenile arthritis and clinically atypical immune dysregulation and / or immunodeficiency.

## Background

LPS responsive beige –like anchor protein (LRBA) deficiency is a primary immunodeficiency disease that was first described by Lopez-Herrera G et al. in 2012 [1]. LRBA deficiency has a broad and variable clinical spectrum which includes autoimmune manifestations mainly involving the gastrointestinal, endocrine and hematologic systems. Patients also experience immunodeficiency and recurrent infections [2–4]^.^ Arthritis is a less well-known autoimmune manifestation of LRBA deficiency. We herein present a case of LRBA deficiency which initially presented with polyarticular arthritis and was diagnosed with Juvenile Idiopathic Arthritis (JIA). Additionally, Medline and PubMed literature review was conducted crossing the keywords: LRBA, JIA, arthritis, joint involvement and rheumatic disease.

## Case presentation

**Fig. 1:**
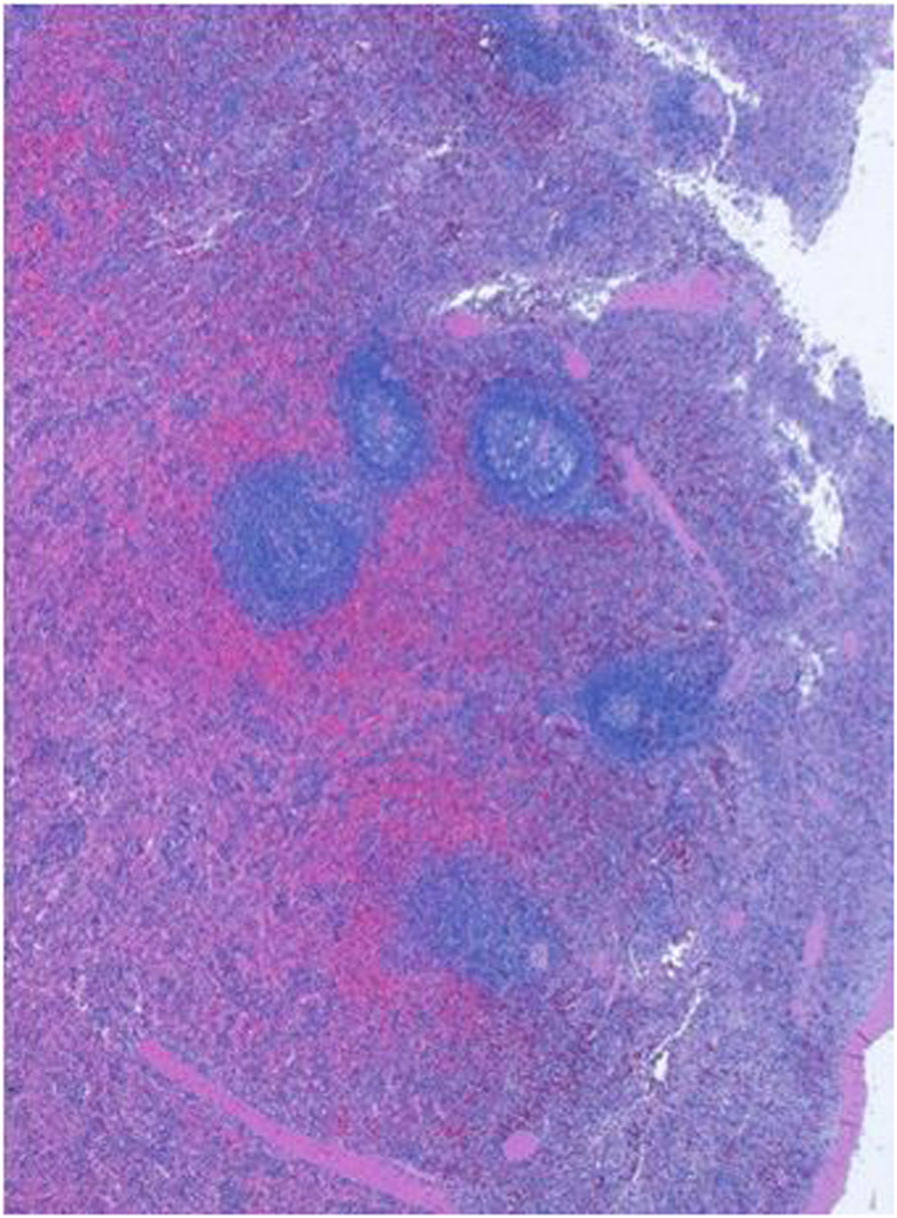
Histological tissue of spleen tissue. Microscopic finding of spleen biopsy specimen shows some expansion of the white pulp and follicles with a few secondary follicles. There is a modest lymphoid infiltrate in the cords and sinuses. This mild lymphoid hyperplasia consists with autoimmune disease

**Fig. 2:**
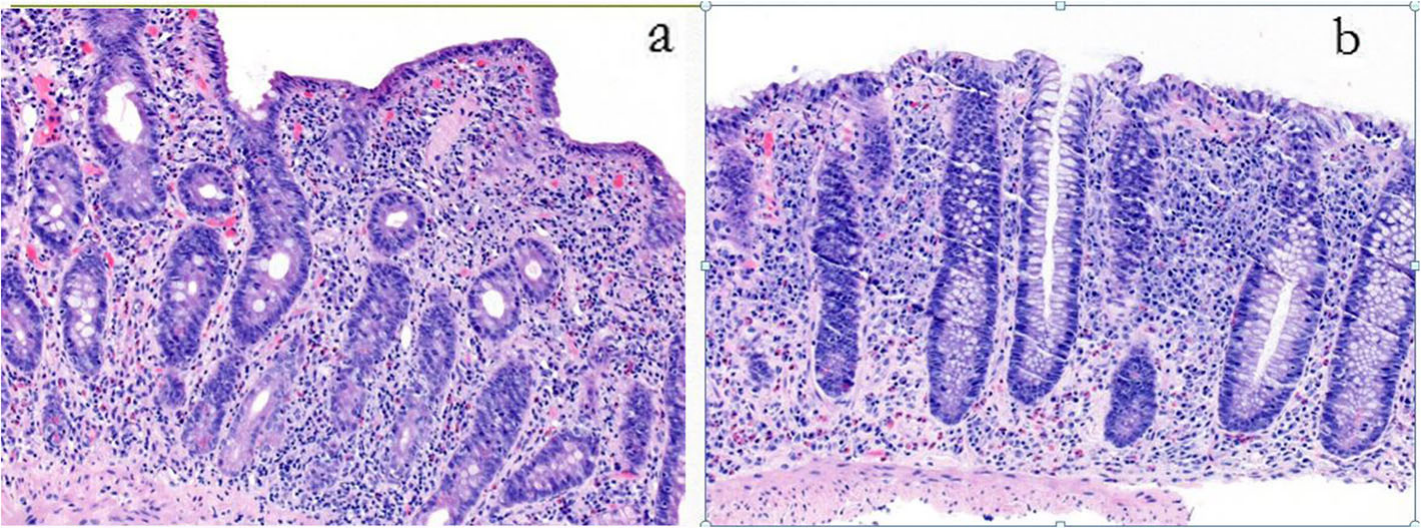
Histological tissue of small and large bowel. Microscopic finding of the duodenum (**a**) shows diffuse duodenitis with severe villous blunting. **b** shows colon histopathology with diffuse lamina propria inflammatory cell infiltrate of lymphocytes and plasma cells with a prominent component of eosinophils and a few scattered admixed neutrophils

**Fig. 3:**
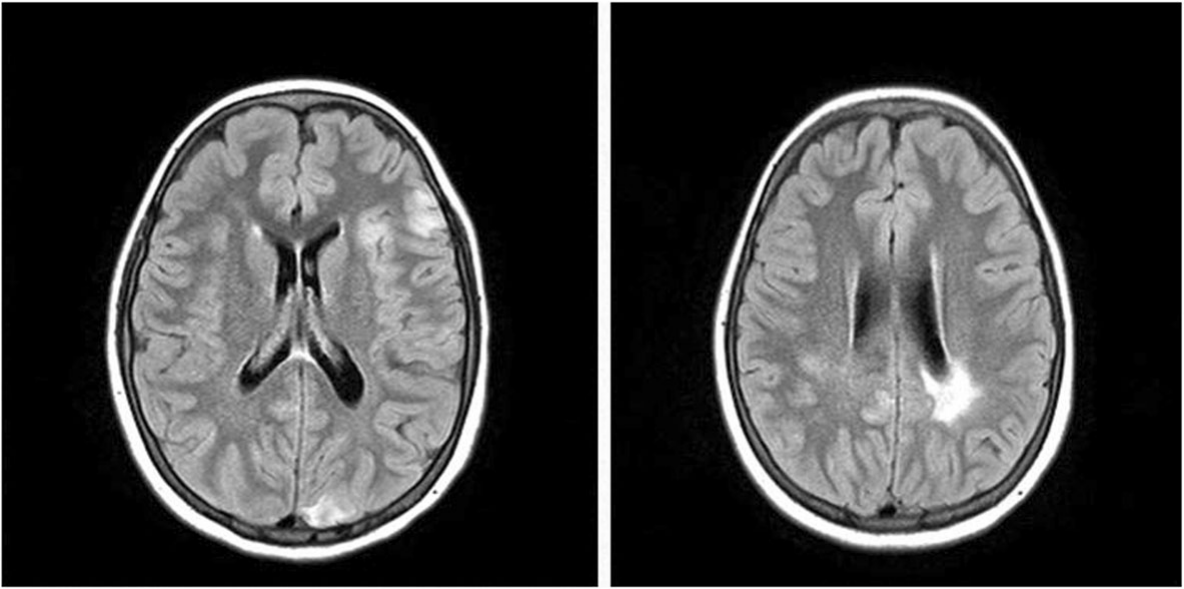
Brain MRI. Brain MRI with multiple scattered abnormal areas of focal T2/FLAIR hyperintensity in the cortex and white matter of bilateral hemispheres and transferred to Comer

**Fig. 4:**
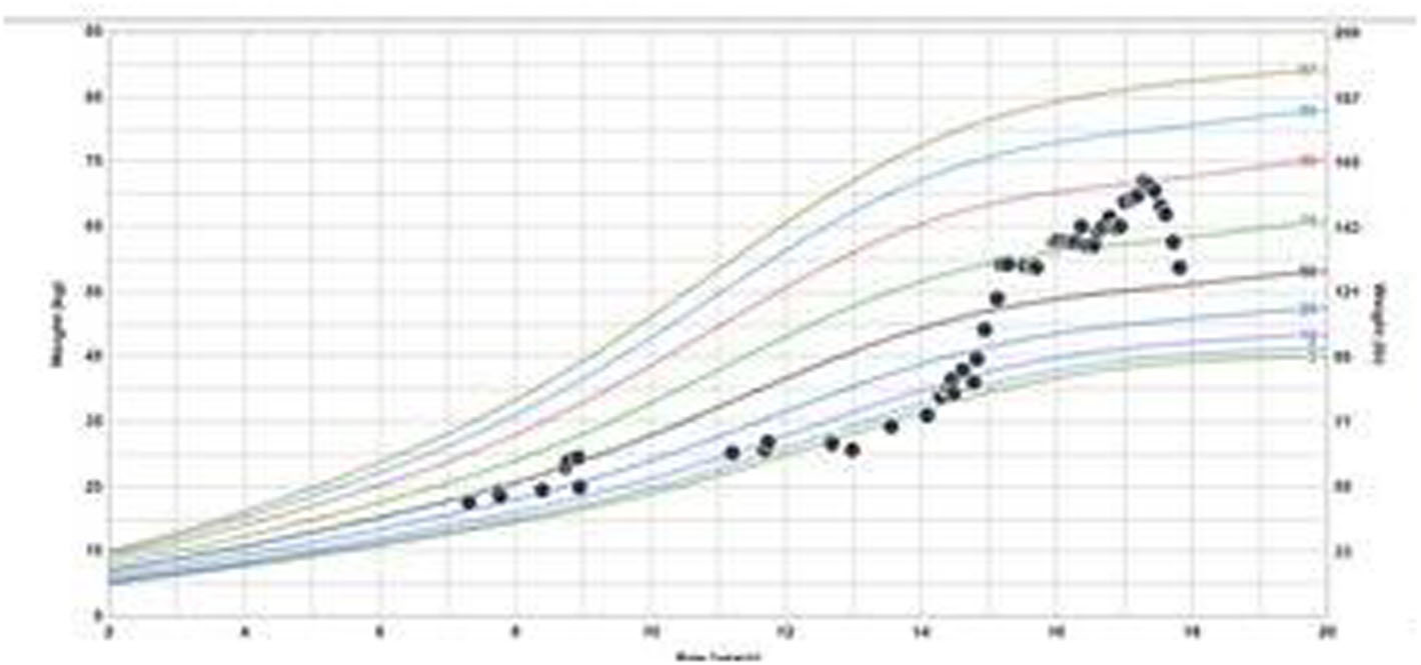
weight growth chart. Weight for age percentile – weight percentile has increased from 3rd percentile to 90th after starting treatment with Abatacept at the age of 15

A 3 year old, Caucasian girl, daughter of non-consanguineous parents with no known relevant family history, initially presented with polyarticular arthritis involving the bilateral knees and ankles, and was diagnosed with juvenile idiopathic arthritis (JIA). During the next few years her joint disease extended to also involve the small joints of the fingers. She responded well to corticosteroid injection of knees and ankles, but arthritis eventually recurred. Over the next several years she was treated with various combinations of corticosteroids, non-steroidal anti-inflammatory (NSAIDS) drugs and methotrexate with inadequate response, and was thus started on etanercept. At age 6, several months after initiation of etanercept, she developed persistent fever of unknown origin, splenomegaly, lymphadenopathy, and autoimmune cytopenias, including clinically mild autoimmune hemolytic anemia (hemoglobin 10.1) and marked neutropenia with absolute neutrophil count of 0.20 K/uL. Etanercept was stopped, and she underwent a thorough immunologic evaluation. Testing for autoimmune lymphoproliferative syndrome (ALPS) via Fas-mediated apoptosis assay was negative. Ferritin was only slightly elevated at 273. Hepatic transaminases and triglycerides were normal. Increased hemophagocytic cells were noted on bone marrow biopsy, along with elevated soluble IL-2 receptor of 9865 U/mL, and decreased number of NK cells (per report, number was not adequate to assess for perforin activity). Thus, early or evolving macrophage activation syndrome (MAS) was suspected, along with ongoing JIA and immune neutropenia. She was treated with corticosteroids and rituximab with temporary resolution of the fevers and immune neutropenia, and improvement in lymphadenopathy and splenomegaly. She subsequently developed recurrent moderate neutropenia, along with severe, refractory autoimmune hemolytic anemia with hemoglobin nadir below 3 g/dL. At this point, the cytopenias failed to respond significantly to immunoglobulin (IVIG), rituximab, cyclosporine and splenectomy (at the age of nine). Spleen pathology showed mild lymphoid hyperplasia consistent with autoimmune disease (Fig. 1). Due to ongoing anemia and neutropenia post splenectomy, treatment with sirolimus was started, with rapid significant improvement and ability to wean steroids. Additional inflammatory features included autoimmune enteritis. Colon biopsy showed diffuse lamina propria inflammatory cell infiltrate of lymphocytes and plasma cells (Fig. 2), pulmonary nodules, and brain lesions (Fig. 3). Diagnostic work up identified compound heterozygote mutation in LRBA. At the age of 15 she started treatment with abatacept, intravenous 440 mg every 60 days, in addition to subcutaneous abatacept every 14 days. She demonstrated significant improvement of her symptoms, growth (Fig. 4) and brain imaging. She continued to have mild arthritis which responded to NSAIDS until the age of 17 when she developed bilateral knee swelling while the subcutaneous abatacept was temporarily on hold due to infection; she was successfully treated with intraarticular steroid injection and restarted the subcutaneous abatacept. She was then maintained on a stable regimen of sirolimus and abatacept until undergoing successful allogenic stem cell transplantation (her unaffected brother was the donor) at age 18 years.

### Literature review

After a stringent selection of previous cases with diagnosis of LRBA deficiency, 43 publications were reviewed. Among these 7 patients were identified with arthritis (Table 1). Age of first presentation was six weeks to 3 years. Male Female ratio was 4/3. Two patients were diagnosed with polyarticular JIA and three with oligoarticular JIA. Each patient was found to have different genomic mutation. The treatment was diverse and included corticosteroids, cyclosporine, methotrexate, adalimumab and abatacept.

**Table 1:**
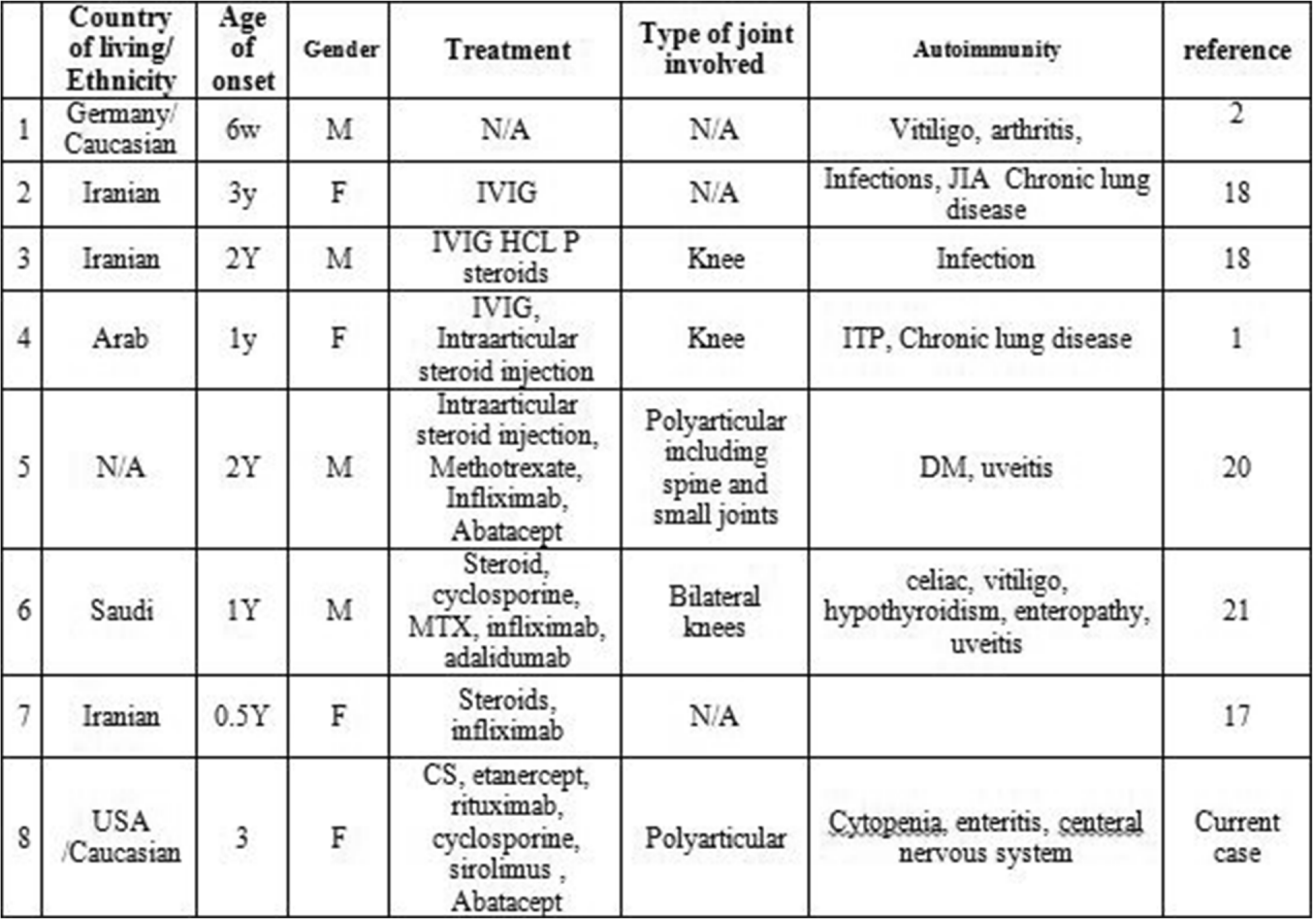
Demographic and clinical data

*CS* Corticosteroids, *IVIG* Intravenous immunoglobulin, *JIA* Juvenile idiopathic arthritis, *ITP* Immune thrombocytopenia, *DM* Diabetes mellitus

## Discussion

Mutation in the LRBA gene was first described by Lopez Herrera et al. who reported four consanguineous families with childhood-onset humoral immune deficiency and features of autoimmunity [1]. These individuals were found to have distinct homozygous mutation in the LRBA gene. The LRBA gene is located on 4q31.3 and encodes the LRBA protein. LRBA is a cytosolic protein that helps to maintain intracellular stores of cytotoxic T-lymphocyte–associated antigen 4 (CTLA-4) protein and prevents its degradation within lysosomes. CTLA-4 is a protein which downregulates T cell immune responses by binding to CD80 and CD86 and transmitting inhibitory signal. It is constitutively expressed in regulatory T cells and is upregulated in conventional T cells after activation [5, 6]Adequate intracellular stores of CTLA-4 are necessary in order for the protein to mobilize quickly to the cell surface and perform its T-cell inhibitory function. Dysfunction of LRBA leads to depletion of intracellular stores of CTLA-4, which causes a functional deficiency of CTLA-4, leading to failure of T cell downregulation and inappropriate T cell activity [7, 8]^.^ Lo et al. demonstrated that total CTLA-4 was substantially depressed in T regulatory cells from an LRBA deficient patient [9]. Interestingly, decreased activity of CTLA-4 may also play a role in JIA. In a 2015 study, children with active JIA demonstrated impaired ability of CTLA-4 to downregulate T cells, despite increased CTLA-4 expression. In this population, CD4 + CD28- cells were increased. These CD28 negative T cells are not susceptible to inactivation by CTLA-4 [10].

LRBA deficiency is categorized within the group of immune dysregulatory disorders clinically classified as common variable immunodeficiency (CVID) [1]. CVID is defined as increased susceptibility to infection or autoimmunity in addition to decreased IgG and IgA and relatively normal T cell levels [11]. An increasing number of patients classified as CVID have had specific genetic mutations identified. The association between rheumatic disease and CVID is well established, with reports of 5–13% rheumatic involvement- mainly inflammatory arthritis- among CVID patients [12–15]. Azizi et al. [16] found 10% prevalence of rheumatic diseases among 227 patients with CVID, with JIA being the most common manifestation (3%). In 35% of these patients- as in our reported patient- the rheumatic disease preceded the diagnosis of CVID.

With regard to LRBA deficiency specifically, the growing number of cases and cohorts reported reveal its highly variable phenotypic expression [2, 3, 17–19], including immune dysregulation, organomegaly and recurrent infection. The primary autoimmune involvement described includes the gastrointestinal system (enteropathy), cytopenias, type I diabetes mellitus, and central nervous system inflammation.

Arthritis is a less frequently reported autoimmune manifestation of LRBA with minimal published data regarding its clinical features and characteristics. Here, we report a patient ultimately diagnosed with LRBA deficiency, who initially presented with JIA. A summary of 14 CTLA-4 deficiency patients show arthritis prevalence of 14% [20].

A thorough search of the literature using PubMed and Medline for data on LRBA deficiency and arthritis revealed details of 7 previously reported patients (Table 1). Alkhairy et al. [20] reviewed the clinical and laboratory features of 31 patients with LRBA deficiency. Eight patients (25%) had arthritis or arthralgia including 2 who were specifically diagnosed with JIA: One female patient was diagnosed with CVID when she was 6 and with JIA when she was 7 yrs. old. She required hospitalization for JIA exacerbation at age 8 and developed septic arthritis of the knee later that year. A male patient developed rheumatoid factor positive, ANA positive knee arthritis at age of 12. His treatment included prednisone and hydroxychloroquine. Lopez Herrera et al. [1] reported a female patient who initially presented with immune thrombocytopenia purpura (ITP) and recurrent infections. She was diagnosed with CVID at the age of 10 and developed knee monoarthritis at age 11, which responded well to intra-articular steroid injection. Levy et al. [21]were the first to describe severe joint disease in a LRBA deficient patient. This patient presented with type I diabetes at 20 months old and with polyarticular JIA and uveitis at the age of 2. The arthritis was described as erosive, affecting multiple joints including cervical spinal and small joints. He had positive ANA and RNP. His treatment included NSAIDS, intraarticular steroids injections, Methotrexate (MTX) and TNF inhibitors as well as steroid eye drops for the uveitis. His treatment was marked by partial response to multiple biologics, with eventual good response to abatacept. Lastly, Mayouf et al. [22] reported a patient with bilateral non erosive arthritis in addition to other autoimmune manifestations – enteropathy, vitiligo, uveitis and hypothyroidism. The patient had partial response to abatacept.

Clearly the limited data reported thus far demonstrate significant variability in age of onset and pattern of joint involvement among LRBA deficient patients with arthritis. One common feature appears to be good response to abatacept [9, 22–24]. Abatacept, primarily used to treat inflammatory arthritis, appears to correct the functional CTLA-4 deficiency seen in LRBA deficiency. Abatacept is a fully human recombinant protein comprising the extracellular domain of human CTLA-4 and a fragment of the Fc portion of human IgG. It binds to CD80 and CD86 thus blocking the interaction with CD28, which is essential for full T cell activation [25–27]. Abatacept appears to have efficacy for multiple autoimmune features of LRBA deficiency including chronic lung disease inflammatory arthritis. There are no established recommendations for the dose or frequency of abatcept for LRBA deficiency patients, previously reported doses were 10–20 mg / kg every one to four weeks [25]. Similarly to the previously reported patients, our patient failed to respond to methotrexate, NSAIDs, etanercept, and rituximab, but responded to abatacept.

The reported patient also showed a robust response to sirolimus. Sirolimus is a macrolide antibiotic with immunosuppressive qualities. It works on B cells and T cells by blocking cytokine-receptor dependent signal transduction [28]. Sirolimus is well known for its effectiveness in treating autoimmune cytopenias [29, 30]. Azizi et al. described the use of sirolimus for multiple drug resistant LRBA enteropathy [31]. Our patient’s life-threatening hemolytic anemia responded rapidly to sirolimus, although she did develop other autoimmune manifestations years later requiring additional therapies.

## Conclusion

Rapid progress in immunogenetics means that patients previously classified as CVID may have a specifically identifiable gene mutation. A portion of these patients present with inflammatory arthritis. Thus, pediatric rheumatologists should consider testing for genetically mediated immune dysregulation syndromes, such as LRBA deficiency, in patients with juvenile arthritis and atypical autoimmunity. Identification of a specific genetic diagnosis can be invaluable both prognostically and with regard to treatment, as in the case of abatacept and sirolimus for LRBA and CTLA-4 deficiencies.

## Data Availability

The datasets used and/or analyzed during the current study are available from the corresponding author on reasonable request.

## Abbreviations

CTLA-4: Cytotoxic T-lymphocyte–associated antigen 4
CVID: Common variable immunodeficiency
IVIG: Intravenous immunoglobulin
JIA: Juvenile idiopathic arthritis JIA
LRBA: LPS responsive beige –like anchor protein
MAS: Macrophage activation syndrome
NSAIDS: Non-steroidal anti-inflammatory
